# Biocontrol potential of mycogenic copper oxide nanoparticles against *Alternaria brassicae*

**DOI:** 10.3389/fchem.2022.966396

**Published:** 2022-08-30

**Authors:** Swati Gaba, Ashutosh Kumar Rai, Ajit Varma, Ram Prasad, Arti Goel

**Affiliations:** ^1^ Amity Institute of Microbial Technology, Amity University, Noida, India; ^2^ Department of Biochemistry, College of Medicine, Imam Abdulrahman Bin Faisal University, Dammam, Saudi Arabia; ^3^ Department of Botany, Mahatma Gandhi Central University, Motihari, BR, India

**Keywords:** *Alternaria blight*, mancozeb, propiconazole, CuO NPs, *Trichoderma*, *Alternaria brassicae*, radial growth, microscopic anlaysis

## Abstract

The biological synthesis of nanoparticles using fungal cultures is a promising and novel tool in nano-biotechnology. The potential culture of *Trichoderma asperellum* (*T. asperellum*) has been used to synthesize copper oxide nanoparticles (CuO NPs) in the current study. The necrotrophic infection in *Brassica* species is caused due to a foliar pathogen *Alternaria brassicae* (*A. brassicae*). Mycogenic copper oxide nanoparticles (M-CuO NPs) were characterized by spectroscopic and microscopic techniques such as UV–visible spectrophotometry (UV–vis), transmission electron microscopy (TEM), scanning electron microscopy (SEM), X-ray diffraction (XRD), and Fourier transform infrared spectroscopy (FTIR). The antifungal potential of CuO NPs was studied against *A. brassicae*. M-CuO NPs exhibited a surface plasmon resonance (SPR) at 303 nm, and XRD confirmed the crystalline phase of NPs. FTIR spectra confirmed the stretching of amide bonds, and the carbonyl bond indicated the presence of enzymes in *T. asperellum* filtrate. SEM and TEM confirmed the spherical shape of M-CuO NPs with an average size of 22 nm. Significant antifungal potential of M-CuO NPs was recorded, as it inhibited the growth of *A. brassicae* up to 92.9% and 80.3% in supplemented media with C-CuO NPs at 200 ppm dose. Mancozeb and propiconazole inhibited the radial growth up to 38.7% and 44.2%. SEM confirmed the morphological changes in hyphae and affected the sporulation pattern. TEM revealed hardly recognizable organelles, abnormal cytoplasmic distribution, and increased vacuolization, and light microscopy confirmed the conidia with reduced diameter and fewer septa after treatment with both types of NPs. Thus, M-CuO NPs served as a promising alternative to fungicides.

## 1 Introduction

In the current scenario, the interdisciplinary field of nanotechnology deals with the synthesis of nanoparticles of different chemical compositions and varying sizes. Due to their small size, metal and metal oxide nanoparticles have been considered promising antifungal and antibacterial agents compared to their bulk equivalents (Singh et al., 2016; Singh et al., 2018). Previous studies proved that biological synthesis enhances the antimicrobial potential of nanoparticles due to the secretion of antimicrobial metabolites, ultimately increasing the efficacy of nanoparticles to 10–20-fold higher as compared to chemically synthesized nanoparticles (Bawskar et al., 2015; Nandini et al., 2017; Singh et al., 2017). Physical and chemical methods of synthesis have limitations on chemical residues, which can be overcome by biological methods (Patra and Baek, 2014). Thus, researchers across the globe are switching to the use of biological systems for the synthesis of nanomaterials.

Over the past few years, the copper compound has been used as fungicides, pesticides, and fertilizers (Ahamed et al., 2014). Several studies examined the toxic effects of CuO NPs on different crops, while their reports suggested that CuO NPs were not toxic up to certain concentrations and efficiently suppressed the disease (Shaheen et al., 2021a; Salem and Fouda, 2021). Thus, to eradicate the facts of toxicity, the current study involves the use of CuO NPs, which have been approved by the Food and Drug Administration (FDA) and the Environmental Protection Agency. The use of CuO NPs has been considered as safe as a 6 mg/kg per hectare dose, which is sufficient for agricultural fields and acts as a biocidal product under the Biocide pesticide regulation (BPR) act existing in European countries (Finckh et al., 2015; Tamm et al., 2015).

CuO has the potential for a wide range of applications; as an antibacterial and antifungal agent exhibiting toxic effects on biological systems, causing the apoptosis of cells (Borkow and Gabbay, 2009; Siddiqui et al., 2013). In the field, these NPs have been widely used as nano-fertilizers, nano-fungicides for the control of plant pathogens, and gene transporters (Dasgupta et al., 2015; Tolaymat et al., 2017). Nano-products increase the efficiency of plant protection products and deliver the fungicides efficiently, have no negative effects to plants at lower doses (Sasson et al., 2007). However, at higher concentrations, traditional fungicides are not safe, as it affects plant health. However, phytopathogens develop resistance against the fungicides (Day et al., 2015).

*Brassica juncea* is an economically important crop that suffers an average yield loss of approximately 10%–70% due to a large number of fungal diseases (Meena et al., 2010). The tremendous yield loss occurred in *Brassica* species per year due to the major fungal disease *Alternaria* blight (IBEF, 2017). The causal organism of the disease is an ascomycete foliar pathogen, *Alternaria brassicae* (*A. brassicae*), that significantly leads to a yield loss of approximately 35%–46% in brassica species (Chattopadhyay, 2008). As per reports, the population of *A. brassicae* possesses resistance against chemical fungicides (Varma et al., 2008). Till date, *Alternaria* blight is managed by various chemical fungicides like mancozeb (0.2%), azoxystrobin (0.05%), propiconazole (0.05%), difenconazole (0.05%), and hexaconazole (0.05%), but the use of fungicides leads to toxicity and affected the oil quality (Singh et al., 2008; Kumar and Rathi, 2014). The existing fungicides have an active constituent of copper; however, their use was neither cost-effective nor safe for the environment.

Williams and cooper (2004) reported in a study that the mixture of copper sulfate and zinc was effective in reducing the mycelial growth inhibition of *A. brassicae*. Thus, it is a need of society to develop long-term, eco-friendly, and cost-effective nano-fungicides. Hence, CuO NPs have been considered as a logical choice for the management of *Alternaria* blight, which hinders the life cycle of pathogenic fungi *A. brassicae*. Also, CuO NPs act as a potent fungicide due to their novel property such as the inhibition of spore germination, ultimately affecting the metabolism of fungi (Khodashenas and Ghorbani, 2014). Thus, considering all the above facts and novel properties of CuO NPs, this study has focused on the mycogenic copper oxide nanoparticles (M-CuO NPs) synthesized from *T. asperellum* filtrate.

Biocontrol agents such as *Trichoderma* species can be used more effectively among different fungal genera since their eco-friendly, non-pathogenic nature has a synergistic effect on the antifungal activity of nanoparticles (Singh et al., 2017). The fungal agents are a natural source of extracellular enzymes and secondary metabolites; thus, these molecules act as a capping and reducing agent for the nanoparticle synthesis. The use of biocontrol agent increases the antimicrobial potential of nanoparticles; in one study, selenium nanoparticles synthesized from *Trichoderma* were more effective and selenium NPs suppressed the pearl millet disease efficiently (Nandini et al., 2017). Interestingly, the saprophytic fungi *T. asperellum* is known for the presence of copper transporters in *T. asperellum,* which helps in the uptake of heavy metal copper (Hoseinzadeh et al., 2017). In addition, the thermophilic adaptation of this microbe secretes an extracellular enzyme at high temperature, possessing several bioactivities that make it a feasible microbe for the synthesis of CuO NPs (Saravanakumar et al., 2016; Stracquadanio et al., 2020). The secondary metabolites like polyketides, peptaibols, terpenes, pyrones, diketopiperazine, phenolics, alkaloids, enzymes, and glycolipids act as reducing agents (Saravanakumar et al., 2015; Rehana et al., 2017).

Therefore, in the present research, M-CuO NPs were synthesized by selecting a microbe *T. asperellum*. To the best of our knowledge, literature studies revealing the antifungal effect of CuO NPs on *A. brassicae* have not been explored yet and the effect of nanomaterial on cytomorphology of the pathogen has not been discussed yet. However, biologically synthesized nanoparticles were more stable and have higher antifungal activity as compared to chemically synthesized copper oxide nanoparticles (C-CuO NPs) (Lee et al., 2011). Hence, in the present study, the biocontrol potential of M-CuO NPs and C-CuO NPs was analyzed and commercial fungicides have been evaluated against a virulent isolate of *A*. *brassicae*.

## 2 Materials and methods

### 2.1 Chemicals and procurement of pathogenic fungal culture

Chemically synthesized copper oxide nanoparticles C-CuO NPs (<50 nm) of CAS No-1317-39-1 were purchased from (Sisco Research Laboratories Pvt. Ltd., Maharashtra, India). The commercially available fungicides Uthane M-45 Mancozeb (75%) and propiconazole (25% EC), Syngenta were purchased from online sources. The culture of *A*. *brassica*e (MZ722977) was obtained from the Division of Plant Pathology, Indian Agricultural Research Institute, New Delhi, India.

### 2.2 Fungal culture maintenance

*T. asperellum* (Indian Type Culture Collection-8011) was obtained from the Division of Plant Pathology, ICAR-Indian Agricultural Research Institute, New Delhi, India. The fungal culture was sub-cultured on potato-dextrose agar (PDA) and kept at 25 ± 2°C for 4–5 days. The culture disc of *T*. *asperellum* from 5 days old culture plate was cut with a cork borer and inoculated into a conical flask containing potato dextrose broth (PDB).

### 2.3 Preparation of filtrate from the wet biomass of fungus

The flask containing PDB inoculated with spores of *T*. *asperellum* was incubated at 25 ± 2°C for 7 days for the growth of fungal biomass. Thereafter, 25 gm of the biomass of *T*. *asperellum* was suspended in sterilized distilled water and the flask was kept at a rotary shaker at 150 rpm at 40–50°C for 24–72 h. Further, the culture filtrate without fungal cells (CFWFC) was separated and filtered through Whatman filter paper no. 1.

### 2.4 Filtrate-mediated mycosynthesized copper oxide nanoparticles

For the synthesis of M-CuO NPs, 50 ml of CFWFC was mixed with 50 ml of copper sulfate solution (CuSO_4_·5H_2_O), 100 mM, and the flask was kept in the rotary shaker at 150 rpm at 40–50°C (Cuevas et al., 2015; Bukhari et al., 2021; Gaba et al., 2022). The conical flask containing the CuSO_4_ solution (Negative control) and the positive control containing the *T*. *asperellum* filtrate were also maintained. A color change was observed in the reaction mixture from yellow to green and finally, a red precipitate of M-CuO NPs was found to be settled at the bottom of the flask after 2.5 h. The mixture was centrifuged at 8,000 rpm for 10 min. The recovered pellets were washed in deionized water. The pellet was kept overnight at 40°C for air drying. Finally, the powder of obtained M-CuO NPs was collected and stored in Eppendorf tubes.

### 2.5 Characterization of copper oxide nanoparticles

#### 2.5.1 UV–vis spectra, DLS, and zeta potential of mycogenic copper oxide nanoparticles

The preliminary characterization of synthesized M-CuO NPs was carried out by a UV–visible spectrophotometer, Cary 100 (Noida, India) in the 200–800 nm range (Hassan et al., 2019). For UV–vis characterization, 10 mg/ml of M-CuO NPs was suspended in water, as this technique characterizes the nanoparticles based on surface plasmon resonance and optical properties.

DLS and zeta potential were used to measure the mean particle size and surface charge on the surface of nanoparticles, respectively. An instrument Malvern Zeta sizer (Nano ZS90, Noida, India) was used to analyze the particle size and zeta potential. For this, the dried powder of M-CuO NPs was suspended in distilled water and sonicated for 2 h. All measurements were carried out in triplicate with a temperature equilibration of 1 min at 25°C with an angle of 90°C (Consolo et al., 2020).

#### 2.5.2 X-ray diffraction analysis and Fourier transform infrared spectroscopy

XRD (D2 Phaser, Model:08, Discover, Bruker, Raipur, India) was used to find out the crystalline structure of the nanoparticles scanned at 10–80° *θ*. FTIR (Shimadzu, Noida, India) was carried out to find out the functional groups in the filtrate of *T*. *asperellum.* The sample of M-CuO NPs was crushed in KBr pellets in a mortar and pestle and scanned for FTIR analysis in the range of 4,000–500 cm^−1^.

#### 2.5.3 Scanning electron microscopy with EDX and a high-resolution transmission electron microscope

The surface morphology of M-CuO NPs was characterized by SEM. For analysis, a thin film of the sample was prepared on the carbon-coated grid and examined under the instrument (Model no-ZEISS EVO 18, Raipur, India). TEM (JEM-1011 EX microscope, Delhi, India) was used for the analysis of the size and particle size distribution of CuO NPs. A drop of M-CuO NPs was placed on a copper grid, and the solvent was evaporated before observation. Then, the sample was examined under TEM.

### 2.6 Stress enzyme analysis of *A. brassicae* treated with copper oxide nanoparticles

Beauchamp and Fridovich (1971) was used to extract superoxide dismutase (EC 1.15.1.1) from fungal mycelium treated with NPs. Baureder et al. (2014) method was used to extract catalase from fungal mycelium treated with CuO NPs. Spectrophotometric determination of antioxidant enzymes was analyzed through (Raghib et al., 2020).

### 2.7 Antifungal activity of copper oxide nanoparticles

The antifungal potential of M-CuO NPs and C-CuO NPs was evaluated using the poisoned food technique at different concentrations (25, 50, 100, 150, and 200 ppm) against *A*. *brassica*e (Ali et al., 2020). In this technique, PDA was mixed with different concentrations of CuO NPs. Simultaneously, plates of two commercial fungicides at their standard concentration served as the positive control, mancozeb (0.2%) and propiconazole (0.05%). PDA plates without nanoparticles served as a negative control. The media were poured into sterile Petri plates, which were further solidified. Thereafter, a 5 mm disc of *A*. *brassicae* from the 10th day old culture plate was placed in the center of each Petri plate. After that, Petri plates were kept in an incubator at 25 ± 2°C until the fungus growth reached the edge of the Petri plates in the negative control. Fungal growth was observed on the 5th, 10th, and 15th days of incubation. Percentage inhibition of fungus mycelium was calculated by the following formula:

Percentage inhibition = [(C-T)]/[C]×100,

where C= control and T = treatment.

### 2.8 Morphological examination in *A. brassicae*

For the analysis of morphological characteristics in *A*. *brassicae*, fungal cultures were grown in Petri plates with PDA mixed with various concentrations of NPs. Petri dishes were incubated for 7 days at 25 ± 2°C. The comparative analysis of conidia collected from the control sample and from each treatment was performed. For size measurement, they were visualized under microscope (Carl Zeiss, Germany) at ×40 magnification, and the dimensions of conidia were measured by randomly selecting 15 conidia from each treatment.

Moreover, shape changes of conidia, horizontal, and vertical septa were also observed. The experiment was repeated thrice and in replicates. Adverse effects were observed in fungal mycelium at 200 ppm after treatment with M-CuO NPs and C-CuO NPs. Therefore, for analysis with SEM and TEM, 200 ppm concentration was selected and further studied.

### 2.9 Analysis of the interaction of nanoparticles with fungal mycelium through SEM/EDX

PDB were mixed with a 200-ppm solution of M-CuO NPs and C-CuO NPs, respectively. The broth mixed with standard fungicides served as positive control and negative control was also maintained. All the conical flasks containing PDB mixed with NPs were autoclaved at 121°C at 15 psi and kept at room temperature for cooling. Then, spore suspension containing (1 × 10^5^ spores/100 ml) of *A*. *brassicae* was inoculated in PDB and the flasks were kept in an incubator at 25 ± 2°C. After the growth of fungal biomass, the supernatant was separated from each flask. Further, the fungal biomass after treatment with standard fungicides and with both types of CuO NPs, respectively, was harvested. Then, the biomass was cut with a cork borer, collected in tubes, and fixed with 2.5% glutaraldehyde at 4°C for 24 h. Subsequently, the sample was washed with 0.1 M phosphate buffer (pH = 7) for 5 min. After that, samples were dehydrated in a graded series of ethanol from 30%, 40%, 50%, 60%, 70%, 80% to 90% for 5 min in each solution. The last step was performed in 100% ethanol for 5 min thrice (Bozzola and Russell, 1992). Dehydrated samples were dried in the oven at 40°C and placed on the gold-coated plate for half an hour before SEM observation (Zeiss Evo 18). The interaction of nanoparticles was checked through SEM analysis.

### 2.10 Transmission electron microscope analysis for the interaction of fungal mycelium with copper oxide nanoparticles

The dehydrated dried hyphae were submitted to AIIMS for block preparation. These dried hyphae were then embedded in epoxy resin and cut into ultrathin sections. Afterward, samples were mounted on a carbon-coated copper grid to examine using TEM [TECNAI 200 KV TEM (Fei, Electron Optics, Delhi)].

### 2.11 Statistical analysis

All the statistical analysis including the results of one independent experiment consisting of three replicates were presented as the mean ± SD values. One-way ANOVA was done with WASP (Web Agri-Statistics package). A value of *p* < 0.05 was considered statistically significant.

## 3 Results

### 3.1 Synthesis and characterization of mycogenic copper oxide nanoparticles

#### 3.1.1 Visual observation of mycogenic copper oxide nanoparticles

**FIGURE 1:**
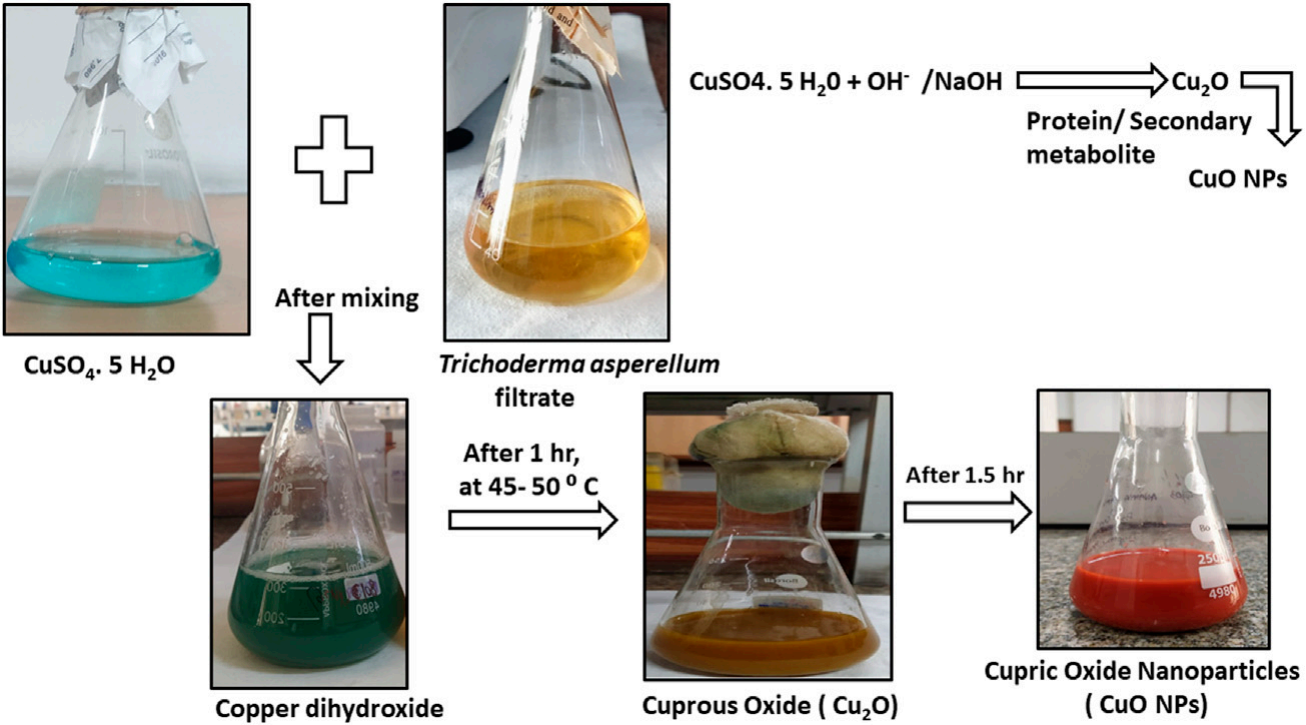
Characterization of M**-**CuO NP synthesis of CuO NPs using culture supernatant of *T. asperellum*.

The synthesis of M-CuO NPs was visualized by the color change of the filtrate. Initially, the color of *T*. *asperellum* filtrate was yellow, which turned into dark green in 10 min at 40–50°C. After 2 h, the dark green color turned into brick red indicating the synthesis of M-CuO NPs (Figure 1). The mechanism of color change in the filtrate is due to a reaction between copper (II) sulfate pentahydrate and hydroxyl anions of water molecules reacted to form copper (II) hydroxide. Thus, the present study is the first report on M-CuO NP synthesis using the filtrate of *T*. *asperellum* in a cost-effective PDB medium within 2.5 h.

#### 3.1.2 Mechanism of synthesized copper oxide nanoparticles by *T. asperellum* culture filtrate

The possible mechanism for the synthesis of CuO NPs could be explained in reference to previous findings and descriptions of several studies reported by Gnanavel et al. (2017) and Nagajyothi et al. (2019). The first step consists of the reduction of Cu^2+^ ion to copper valent state by the hydroxyl anion of water molecules present in the culture filtrate. Generally, the hydroxyl anion of water molecules in the filtrate acts as a reducing agent and forms an intermediate state copper dihydroxide [Cu (OH)_2_].

After that, in the second step, active metabolites and proteins present in microbial cell free extracts convert the Cu (OH)_2_ form into Cu_2_O form. As *T*. *asperellum* species produce active molecules that were predominantly responsible for the M-CuO NPs. The third step consists of heating Cu_2_O at 50°C when hydrogen gets removed in the form of copper hydroxide. After a prolonged reaction rate, stable copper oxide nanoparticles were formed. Thus, microbial compounds such as metabolites act as reducing agents and proteins act as capping and stabilizing agents (Baoshun et al., 2016).

$${\text{CuSO}}_{4}{\text{.}5\text{H}}_{2}\text{O }+2\text{ NaOH}\Rightarrow {\text{ Na}}_{2}{\text{SO}}_{4}+\text{Cu}{\left(\text{OH}\right)}_{2}{+5\text{H}}_{2}\text{O}$$ (A.1)

$$\text{Cu }{\left(\text{OH}\right)}_{2}+\text{Protein/Secondary metabolite}\Rightarrow {\text{Cu}}_{2}\text{O}+\text{By}\;{\text{product}+\text{H}}_{2}\text{O}$$ (A.2)

$${\mathrm{Cu}}_{2}O+{\mathrm{OH}}^{-}+{\text{H}}_{2}O\Rightarrow \mathrm{Cu}{\left(\mathrm{OH}\right)}_{2}{]}^{-}+\mathrm{Cu}\;\mathrm{OH}$$ (A.3)

$$4\;\text{Cu}\;{\text{OH }+\text{O}}_{2}\Rightarrow 4\;\text{CuO}+\;2\;\text{NaOH}$$ (A.4)

### 3.2 UV–visible results

**FIGURE 2:**
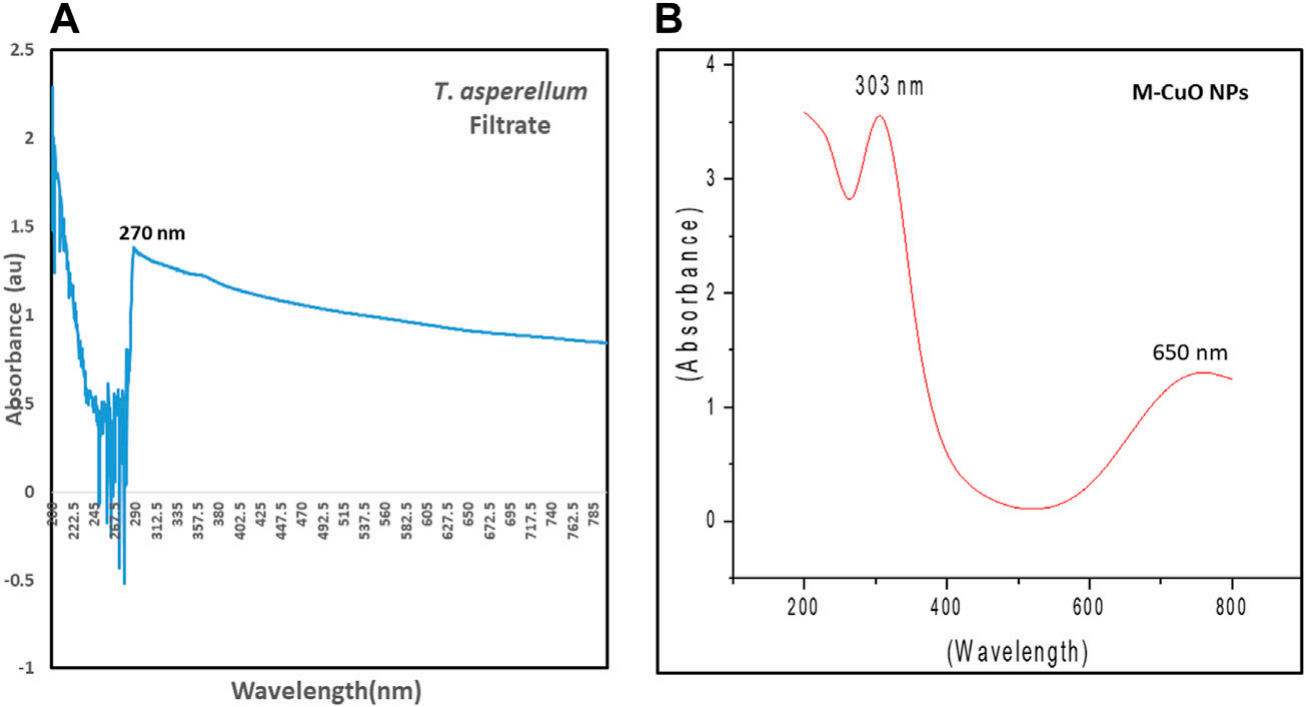
UV–visible spectra of **(A)** *T*. *asperellum* filtrate and **(B)** M**-**CuO NPs.

Spectroscopic studies were monitored for the *T*. *asperellum* filtrate and M-CuO NPs. The filtrate of *T*. *asperellum* indicated a sharp absorption peak at 270 nm at 2.1 lower intensity (Figure 2A). The powder of M-CuO NPs (10 mg/ml) was suspended in water, and the absorption peaks in the ultraviolet range at 300–390 nm with 3.5 intensity and the other peak in the visible range at 620–800 nm with an intensity of 1.0 were recorded (Figure 2B). Hesham et al. (2021) revealed the peak of 300 nm for CuO NPs synthesized from cell-free supernatants of *Pseudomonas fluorescens*. Tiwari et al. (2016) also obtained absorption at a wavelength in the 300–350 nm range and another peak at 570–630 nm of CuO NPs synthesized from cell-free filtrate of *Bacillus cerus* SWSD1. Thus, several studies have suggested that CuO NPs give the absorption peak in the 300–350 nm and in 600–650 nm.

### 3.3 X-ray diffraction studies of mycogenic copper oxide nanoparticles

**FIGURE 3:**
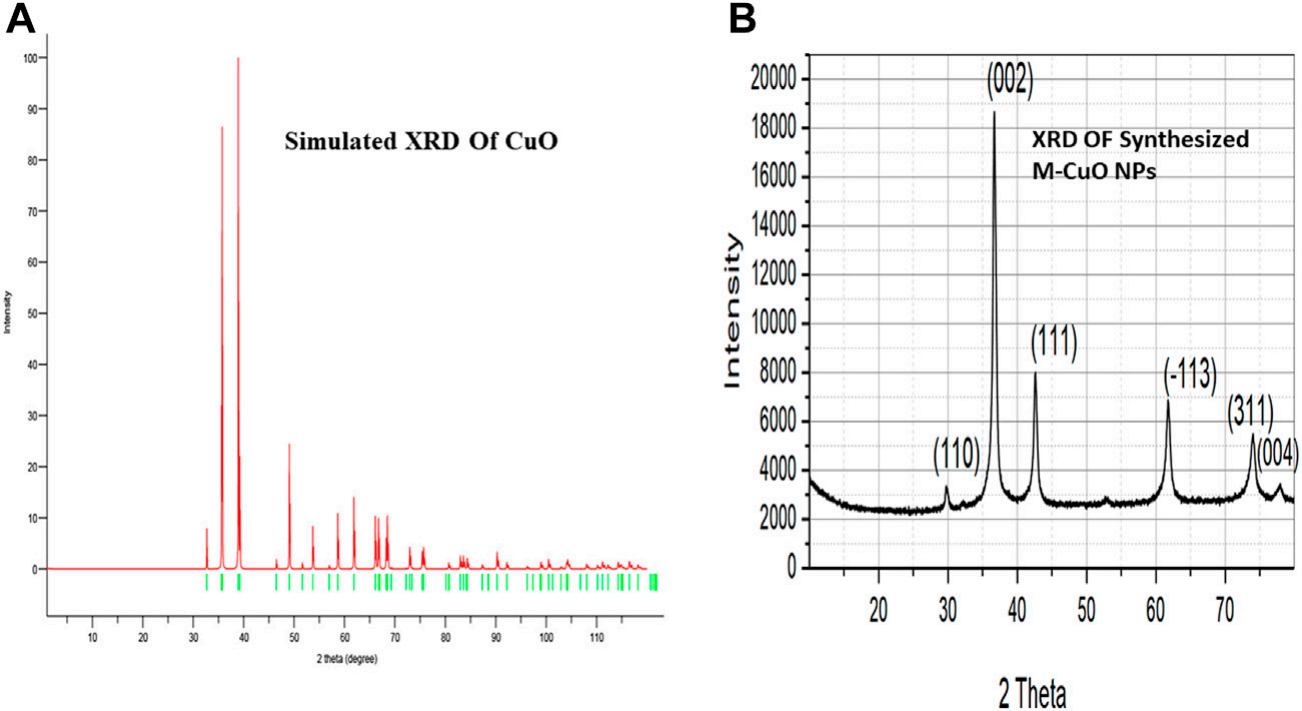
**(A)**. Simulated XRD of M-CuO NPs. **(B)**. XRD of Synthesized M-CuO NPs.

XRD delivers an understanding of the crystal phase of nanoparticles. XRD of simulated CuO is shown in (Figure 3A). XRD diffraction peaks were found at 29.8°, 36.7°, 42.6°, 61.8°, 73.6°, and 77.9°, which is matched with the Joint committee on powder diffraction standards (48–1,584) and planes were assigned (110), (002), (111), (−113), (311), and (004), respectively. The diffraction peaks in (Figure 3B) indicated that the M-CuO NPs have a monoclinic phase. Khatami et al. (2019) have also obtained four strong absorbent peaks for CuO NPs synthesized from *T*. *asperellum* at angles of 29°, 37°, 44°, and 62°. The absence of other sharp peaks confirmed the purity of the sample. The unambiguous background noise was due to the shell of protein around the nanoparticles. A highly intense peak at (002) in contrast to other peaks is the characteristic peak allocated to CuO NPs. Full width at half-maximum (FWHM) values are inversely proportional to the size of nanoparticles. The average crystallite size of M-CuO NPs was 17.5 nm.

### 3.4 DLS and zeta potential

**FIGURE 4:**
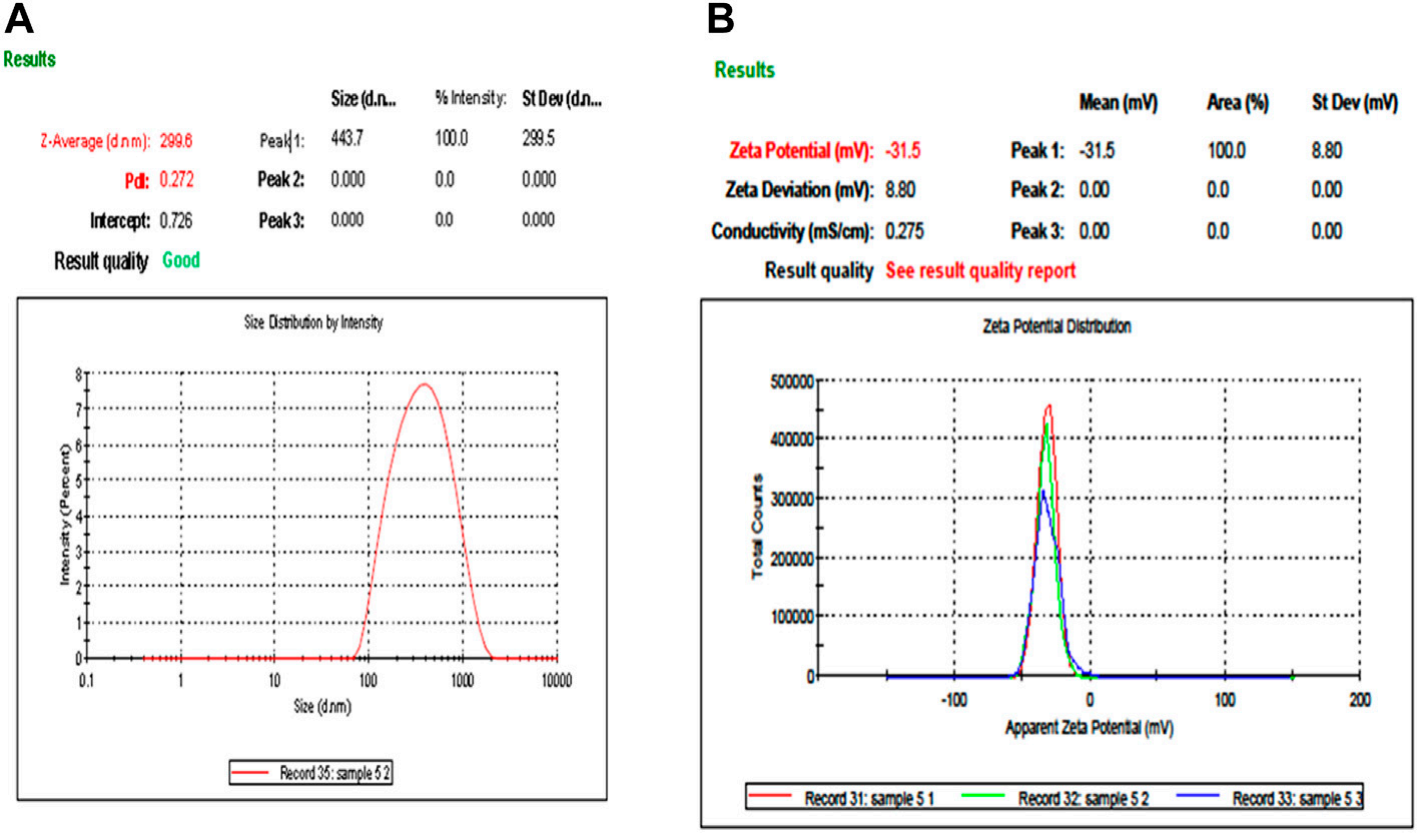
Particle size analysis and stability of M**-**CuO NPs: **(A)** DLS of M**-**CuO NPs and **(B)** Zeta potential of M-CuO NPs.

DLS and zeta potential were used to measure the mean particle size and charge on the surface of nanoparticles, respectively. DLS results were based on the hydrodynamic diameter of NPs synthesized in which NPs were dissolved in a dispersant and responsible for forming noncovalent interactions, causing the particle size to be bigger than SEM and TEM techniques. DLS has given the average particle size of CuO NPs as 299.5 nm, while its hydrodynamic diameter recorded as 499 ± 299.5 nm with a polydispersity index of 0.2882 represents the highly monodisperse nature of nanoparticles (Figure 4A). The zeta potential of CuO NPs was −31.5 mV ± 8.1 (Figure 4B).

### 3.5 Scanning electron microscopy with EDX

**FIGURE 5:**
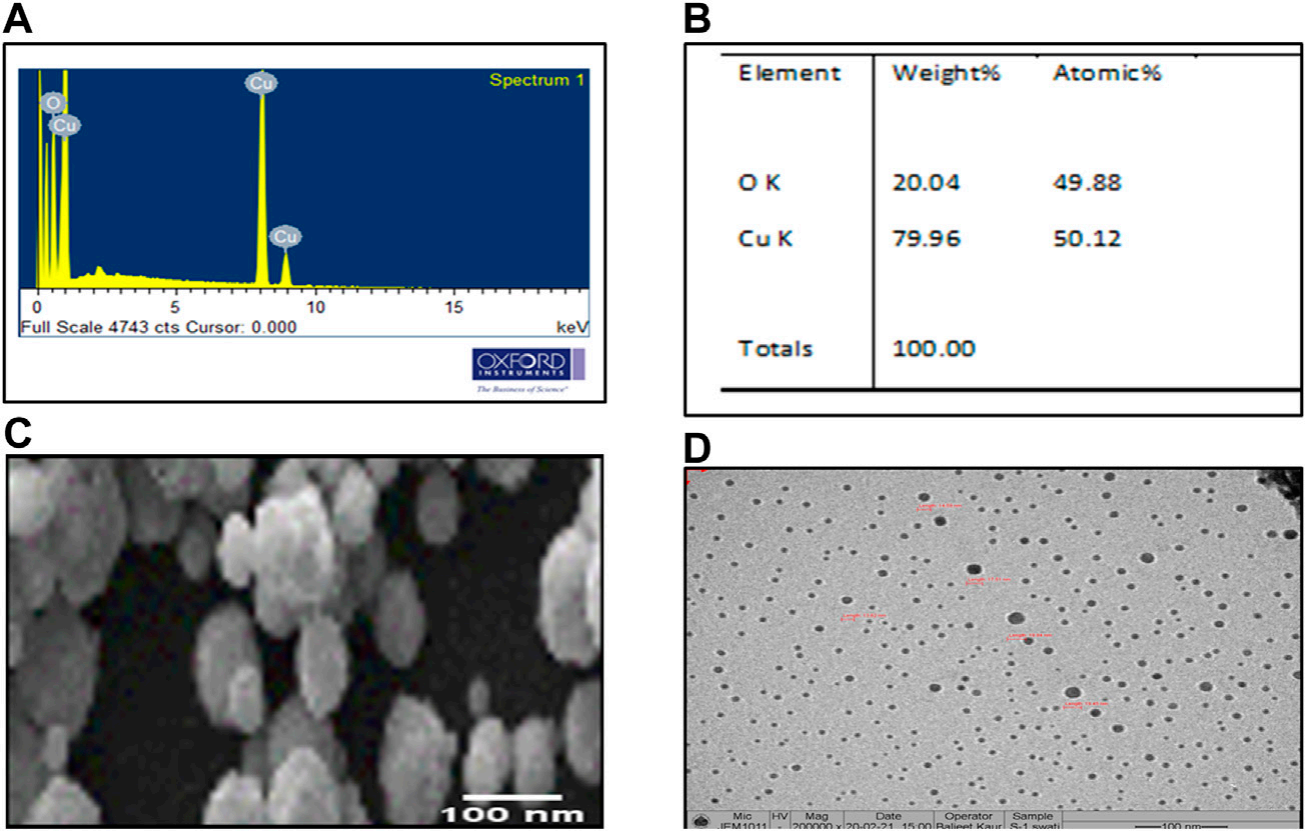
Microscopic analysis of M-CuO NPs: **(A,B)** EDX analysis of M**-**CuO NPs, **(C)** SEM image of M**-**CuO NPs, and **(D)** HR**-**TEM image of M**-**CuO NPs.

EDX analysis indicated the presence of oxygen and copper by 20.04% and 79.96%, weight, respectively. The atomic percentage of oxygen and copper was 49.88% and 50.12%, respectively (Figures 5A,B). The optical absorption band was recorded in the range from 1 to 9 keV. However, M-CuO NPs displayed a peak at 8 keV indicating the surface plasmon resonance of CuO crystallites. These results were matched with the findings of CuO NPs synthesized from *Streptomyces* sp. MHM38 (Maqbool et al., 2018). SEM micrographs of M-CuO NPs confirmed the formation of regular, monodisperse, and spherical shaped nanoparticles (Figure 5C). Gnanavel et al. (2017) have also reported spherical, dense, and agglomerated biosynthesized CuO NPs from *T. asperellum,* which has supported the findings of our M-CuO NPs. As per literature studies, CuO NPs synthesized from different plant species were obtained in spherical and cube shapes (Patra et al., 2012). The particle size distribution of NPs was analyzed by using Image J software by randomly taking measurements of 15 particles and its average diameter was calculated in the 48–56 nm range.

### 3.6 Transmission electron microscope analysis

Particle size distribution of M-CuO NPs was measured by TEM. It was found that M-CuO NPs were monodisperse with a size range from 18 to 22 nm (Figure 5D). TEM results are based on the scattering of electrons in sample irradiation. TEM results showed the core size of NPs, Moreover, particle size distribution estimated in TEM image indicated a majority of the particles in 4–18 nm and 18–22 nm size ranges. As compared to DLS results, where 90.8% intensity of the particles have 333.1 nm size particles and 9.2% intensity of the particles have above 1,000 nm sized particles. Thus, comparatively larger particle size was recorded in DLS due to the hydrodynamic diameter of particles, as compared to the real core size of the particles observed in TEM image, as shown in (Figures 7A, B).

### 3.7 Fourier transform infrared spectroscopy-based characterization of mycogenic copper oxide nanoparticles

**FIGURE 6:**
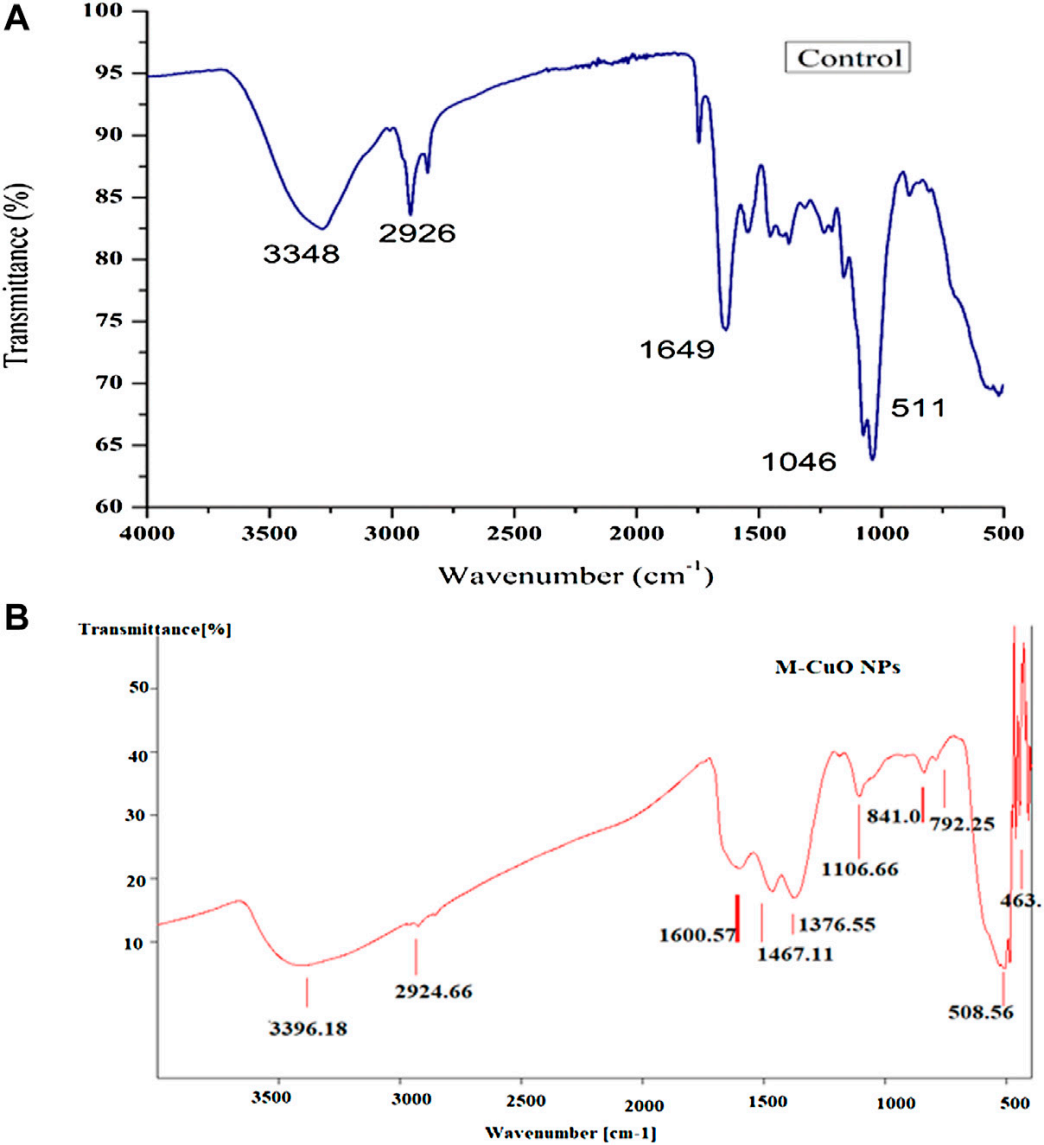
**(A)** FTIR spectra of control and **(B)** FTIR spectra of M**-**CuO NPs.

**FIGURE 7:**
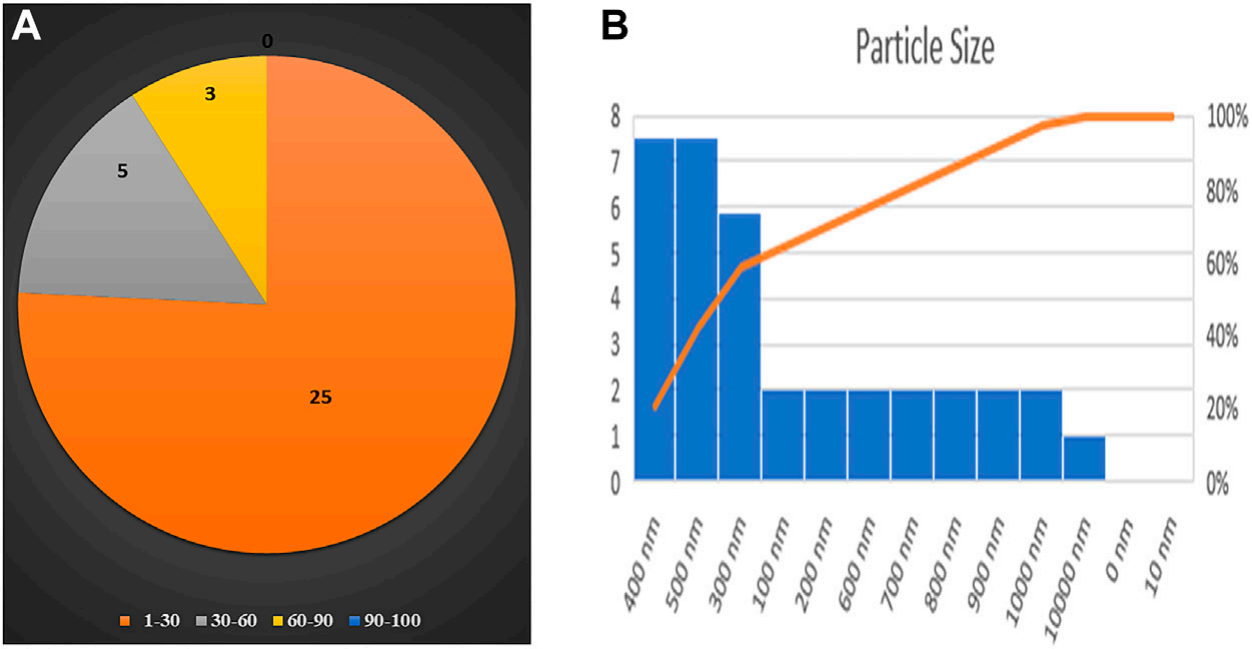
Particle size distribution of M**-**CuO NPs on the basis of **(A)** TEM and **(B)** DLS.

FTIR analysis predicted the presence of functional groups responsible for the reduction and capping of nanoparticle synthesized based on the vibration of different types of bonds present, which absorbs light at different frequencies, when infrared light passes through the sample. The analysis of control (dried powder of *T. asperellum*) showed the stretching of the N–H bonds, C–H bonds of alkane groups, alkyl amine bonds, and the stretching of C=O bonds. However, the aromatic compounds were also predicted, which could be present in phenols and alkaloids, etc., and proteins in the supernatant of *T*. *asperellum* were responsible for the reduction of copper sulfate salt and the capping of M-CuO NPs (Figure 6A; Supplementary Table S1). Thus, the presence of secondary metabolites and proteins in the culture supernatant of *T. asperellum* was responsible for the reduction of copper sulfate salt and the capping of M-CuO NPs. In (Figures 6A,B), the spectrum of *T*. *asperellum* filtrate and M-CuO NPs synthesized is shown in Figure 7B. Spectra of M-CuO NPs indicated the N–H stretch of amide bond, C–H bonds of alkanes, and Cu–O vibrations. Moreover, nitrosamine bonds and C=O stretching were also observed in M-CuO NPs.

### 3.8 Antifungal potential of copper oxide nanoparticles

**FIGURE 8:**
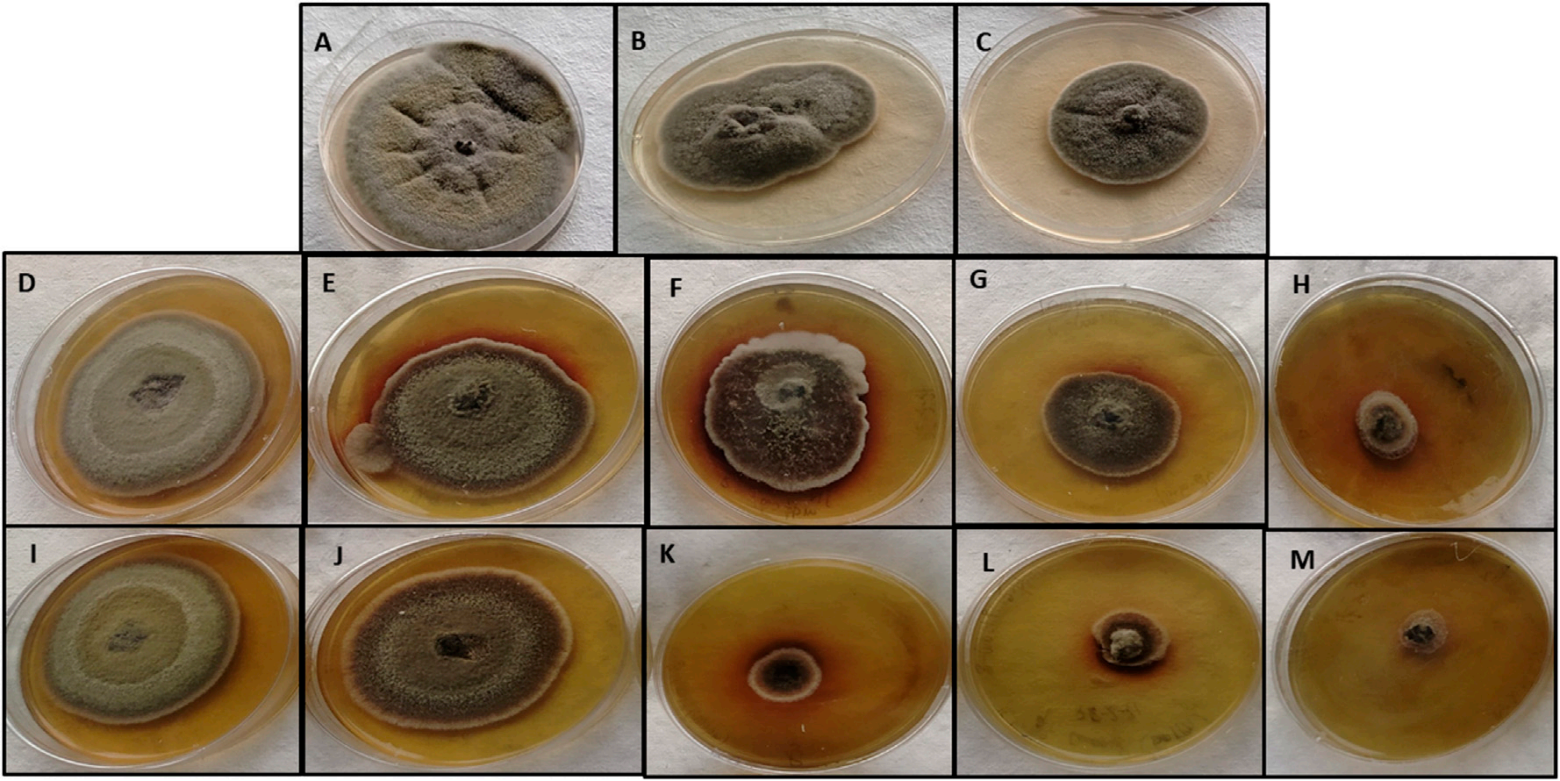
Antifungal activity of M-CuO NPs and C**-**CuO NPs **(A,B,C)**: control, Mancozeb (0.2%) and Propiconazole (0.05%); **(D–H)** C-CuO NPs**—**25, 50, 100, 150, and 200 ppm; **(I–M)** M**-** CuO NPs**—**25, 50, 100, 150, and 200 ppm.

The antifungal activity of M-CuO NPs and C-CuO NPs was analyzed at five concentrations on PDA through the mycelial growth inhibition (MGI) assay, which is considered an efficient and reliable method for determining the fungi toxic action of nanomaterials (Angele Martinez et al., 2017). Significant observations in MGI were measured on the 15th day of incubation due to the slow-growing nature of tested fungi**.** Radial growth (diameter of the colony) of *A*. *brassicae* was measured as 43.3 ± 5.5, 37 ± 8.1, 27.6 ± 4.9, 18.6 ± 6.08, and 5.1 ± 0.1 mm, respectively, at 25, 50, 100, 150, and 200 ppm in M-CuO NP supplemented media. Similarly, the radial growth was recorded as 45.6 ± 1.1, 46.1 ± 1, 39.6 ± 5.5, 20.6 ± 1.1, and 14.4 ± 0.3 mm, respectively, in C-CuO NP supplemented media. The complete inhibition of the colony was observed in media mixed with M-CuO NPs at 200 ppm. However, with an increase in the concentration of both types of nanoparticles, a significant reduction in the growth of *A*. *brassicae* was observed (Figures 8A–M).

As compared to the antifungal activity of both types of CuO NPs, fungicides mancozeb and propiconazole reduced the growth to 44.3 ± 4.04 and 40.3 ± 0.5 mm, respectively. As a result, the antifungal assay suggested that fungicides at their higher concentrations were able to inhibit the growth by less than 50%. However, commercial fungicide 2 (propiconazole) and mancozeb inhibited the growth up to 44.2% and 38.7%, respectively (Supplementary Table S2). Thus, it can be concluded that the higher dose of fungicide was less promising as compared to both types of CuO NPs. Among all the treatments compared, M-CuO NPs were found to be most effective, as they inhibited more than 90% of radial growth. After that, C-CuO NPs were effective and occupied the second rank, as they inhibited the growth up to 80%.

It has been found in several studies that copper-based nanoparticles have antifungal potential against various pathogens, which are summarized in Supplementary Table S3. Till date, CuO NPs have been synthesized through various plant extracts, fungal and bacterial cultures such as *Malus domestica, Azadirachta indica,* and *Eicchornia,* etc. (Ahmad et al., 2014; Vanathi et al., 2016; Choudhary et al., 2017). Plant extract-mediated CuO NPs have minimum inhibitory concentration (MIC) and minimum fungicidal concentration (MFC) at 25, 100, and 1000 mg/ml. Moreover, the fungal filtrate-synthesized CuO NPs were found to be efficient at 25–250 ppm dose (Giannousi et al., 2013; Vera-Reyes et al., 2019). Thus, all these studies have showed that lower doses of CuO NPs (25–1,000 ppm) are sufficient to kill the pathogen, which are supporting the antifungal activity of our M-CuO NPs (25–200 ppm).

**FIGURE 9:**
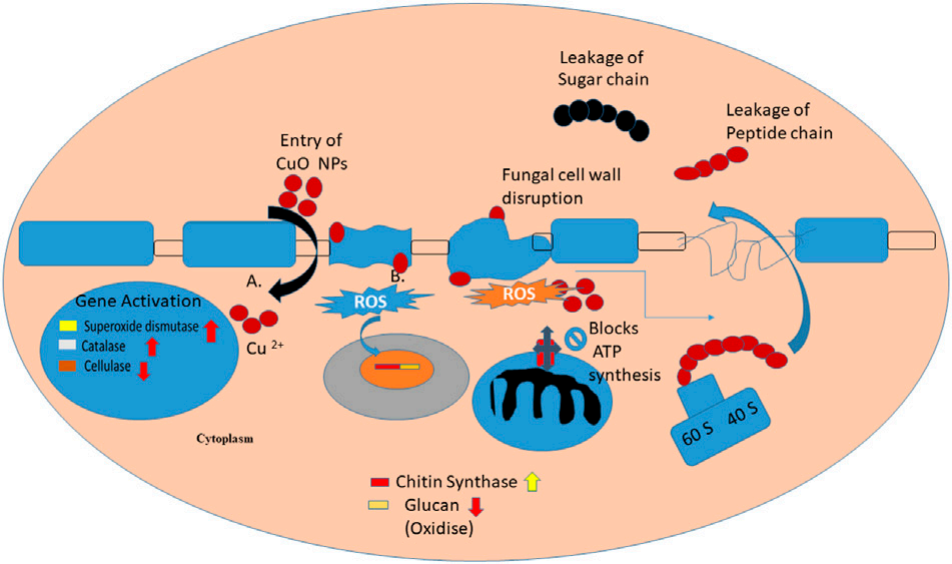
Proposed mechanism of CuO NPs.

The assumed antifungal mechanism proposed through previous studies that CuO NPs generate reactive oxygen species (ROS) *via* mechanisms like Haber–Weiss and Fenton-like reactions, as they entered the cell wall of fungus or dissolved copper ions were responsible for its contact killing mechanism (Figure 9). Fungal cell walls dissolve copper ions, which oxidize the primary components of fungal cell walls, such as glucan and chitin, ultimately causing cell wall disintegration. However, the respiratory metabolism of fungus also gets affected and the expression of chitin synthase genes was downregulated, which is responsible for further morphological changes like irregular fractures, hyphal deformation, uneven swelling, and shrinkage (Fu et al., 2014).

### 3.9 Morphological changes in *A. brassicae* after treatment with copper oxide nanoparticles and fungicides

#### 3.9.1 Effect of mycogenic copper oxide nanoparticles on the conidia length and width of *A. brassicae*

**FIGURE 10:**
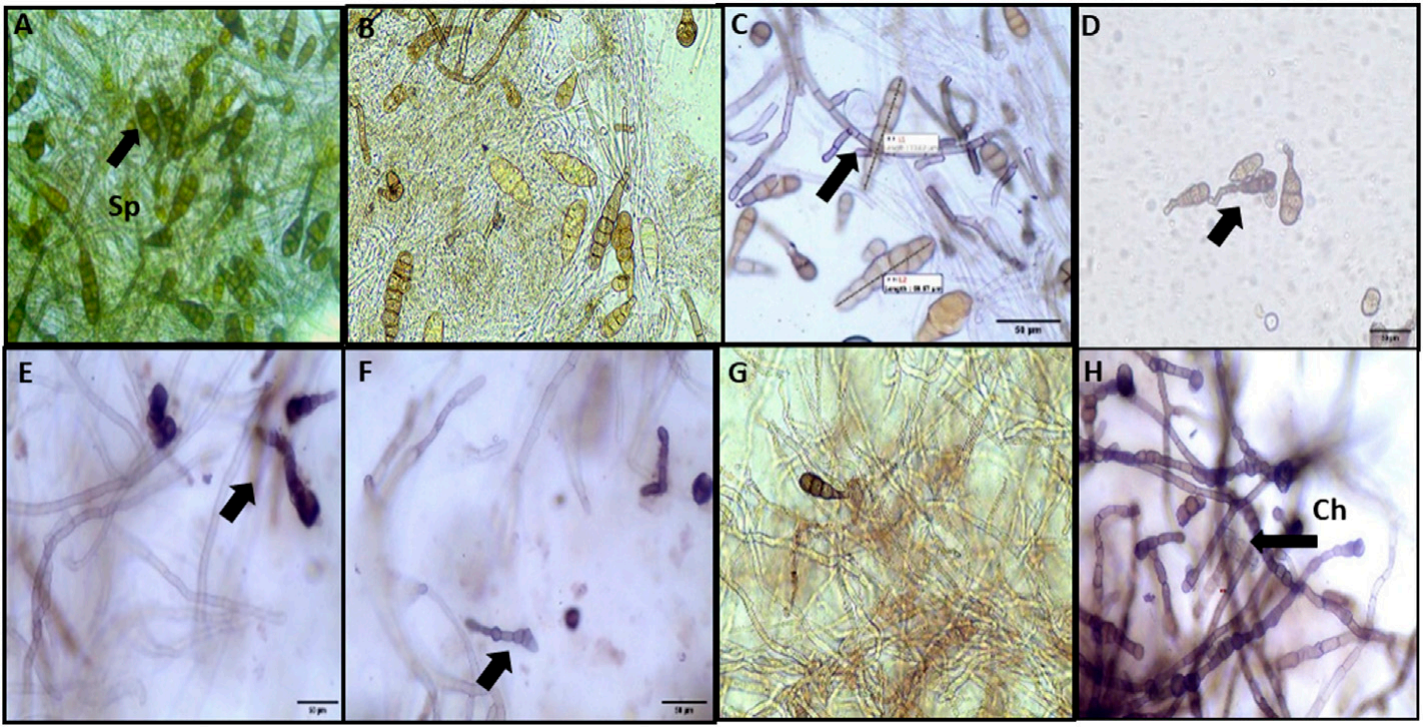
Microscopic observations of the effect of M**-**CuO NPs on the morphology of *A. brassicae*: **(A)** control: long obpyriform conidia; **(B,C)** 25 and 50 ppm: no significant change in the shape of conidia; **(D–F)** 100, 150 ppm, and 200 ppm: bending in the beak of conidia; **(G)** mancozeb: fractures in hyphae; and **(H)** propiconazole: chlamydospore (Ch) formation in hyphae and collapsed conidia.

The morphological characterization of conidia of *A. brassicae* has been studied. The average conidial length in the control sample was varying from 122.2 to 136.8 μm, whereas the conidial width was measured in the range of 40–50 μm. In the control, conidia were abundant and lengthy obpyriform shapes, as shown in (Figure 10A). In both agar plates and broth assays, no significant effect was observed at 25 ppm (Figure 10B), while in mycelium treated with M-CuO NPs, the conidial length was found to be lowered in the range of 122.2–91.0 μm (*p* value < 0.005) and conidial width was decreased to 22.7–16.4 μm (*p* value < 0.005), respectively, from 50 to 200 ppm dose; moreover, significant effects like bending in the beaks of conidia were observed from 100 to 200 ppm (Figures 10C–F; Supplementary Table S4). Horizontal septa were reduced to 4–5 in number as compared to the control group (8–9), and vertical septa were also reduced to 1–2 in comparison to the control group (2–3).

Although from 100 ppm dose hyphae were deformed, shrinkage and irregular swelling were also observed. After treatment with M-CuO NPs, the maximum detrimental effects were detected at 200 ppm, which substantially lowered spore counts and caused the hyphal wall to lose its integrity (Figure 10F). Similar observations were reported by Dhiman et al. (2021). In their study, a comparative study of biologically and chemically synthesized zinc oxide nanoparticles was done, which have reduced the average dimensions of conidia length and conidia width of *A. brassicae* infecting *brassica* species. All these observations have supported the antifungal effects of CuO NPs synthesized in our study.

#### 3.9.2 Effect of fungicides on the conidial length and width of *A. brassicae*

In comparison to control, mancozeb reduced the spore count, the number of septa, conidial length, and width to 96.3 μm × 26.0 μm (*p* value < 0.005). In hyphae treated with mancozeb at 0.2%, irregular swelling and shrinkage were observed (Figure 10G). Whereas propiconazole treatment caused intercalary chlamydospore formation in hypha, a low number of conidia, and reduced conidial length and width (96.2 μm × 23.8 μm) (Figure 10H).

#### 3.9.3 Effect of chemically synthesized copper oxide nanoparticles on the conidial length and width of *A. brassicae*

**FIGURE 11:**
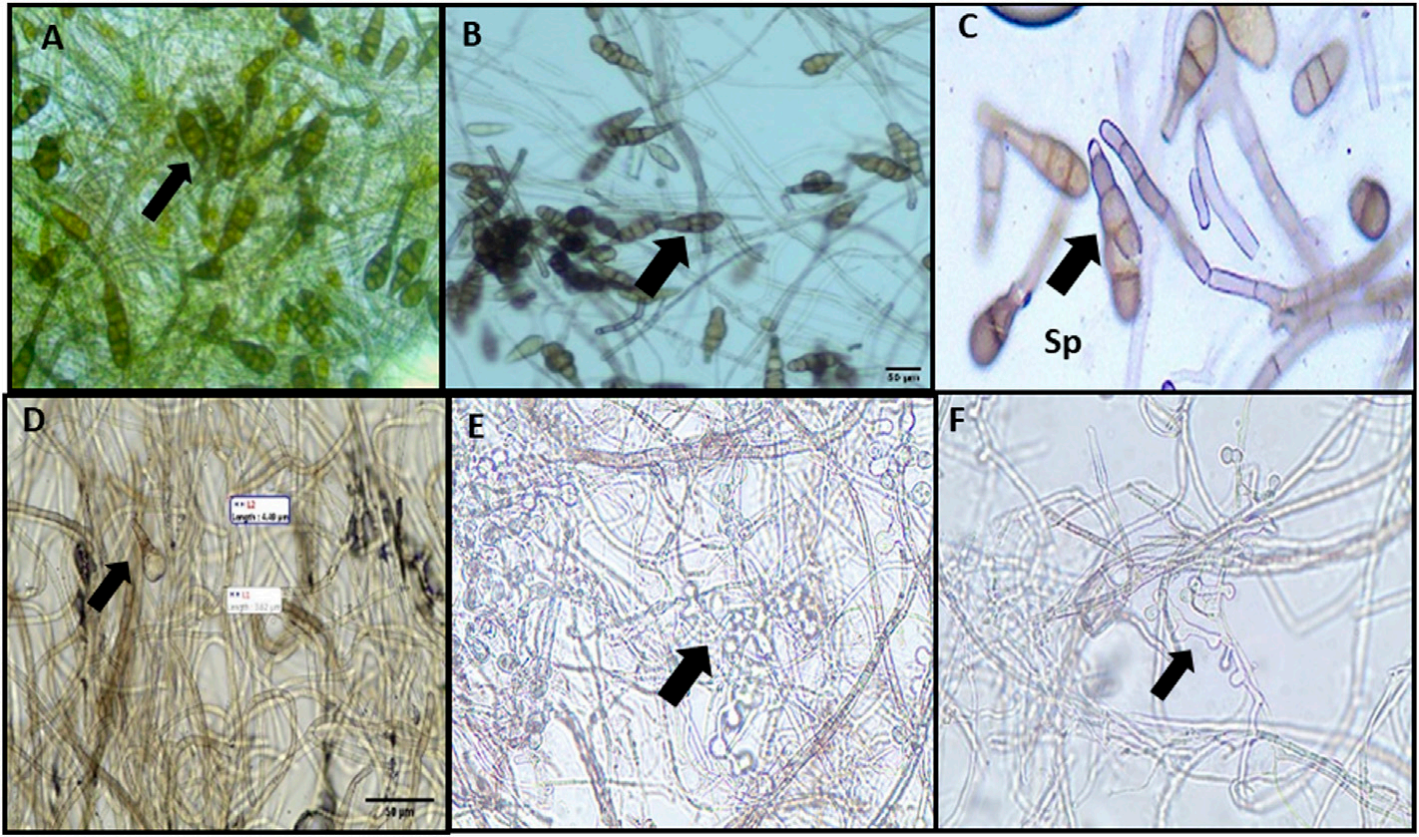
Effect of C**-**CuO NPs on the morphology of *A. brassicae:* **(A)** control: septated hyphae and long obpyriform conidia; **(B)** 25 ppm: long obpyriform conidia; **(C)** 50 ppm: less horizontal septa were observed in conidia; and **(D–F)** 100–200 ppm: less horizontal septation in conidia and spore decreased.

The average conidial length and width of *A. brassicae* were 122.2 to 136.8 μm and 40–50 μm, respectively (Figure 11A). Similarly, C-CuO NPs reduced the conidial length from 105.9 to 99.3 μm (*p* value < 0.005) and conidial width from 36.0 to 35.2 μm (*p* value < 0.005), respectively, from 50 to 200 ppm dose. However, fungal cell walls after treatment with C-CuO NPs produced less significant changes in *A*. *brassicae* morphology (Figures 11B–F). The probable reason for the less significant effects of C-CuO NPs on the morphology of *A*. *brassicae* is the absence of antimicrobial metabolites. Moreover, C-CuO NPs have lower size range particles < 50 nm. Whereas in the suspension of M-CuO NPs, the particle size was in the range of 4–18 nm and 18–22 nm. Thus, the enhanced antimicrobial activity was due to the presence of a mixture of particle sizes and the release of antimicrobial compounds in the filtrate. As a result, however, both types of CuO NPs and fungicides have significant effects; however, the spore germination rate was drastically reduced with M-CuO NPs within 5 days of incubation as compared to other treatments, thus considering this fact, the pathogenicity of culture was lost in M-CuO NPs treatments.

### 3.10 Scanning electron microscope analysis for the interaction of copper oxide nanoparticles with *A. brassicae*

#### 3.10.1 Morphological changes in conidia and hyphae

**FIGURE 12:**
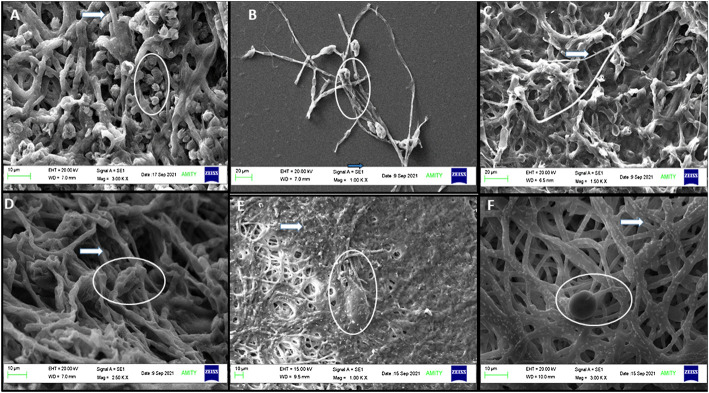
SEM images of *A. brassicae* after treatment with M and C**-**CuO NPs: **(A,B)** control: long obpyriform conidia and maximum sporulation observed at 10 μM and at 20 μM; **(C)** *A. brassicae* after treatment with mancozeb (0.2%); **(D)** fractures were generated in hyphae after treatment with propiconazole (0.05%); **(E)** M**-**CuO NPs (200 ppm): deformed spores formed and disintegrated fragments with fractures in hyphae; and **(F)** C**-**CuO NPs: less spore and NP attachment on hyphal wall. All the above images showed conidia in circles and hyphae with arrows.

The direct interaction of nanoparticles on biological samples, including fungi, virus, and bacteria, showed surface adhesion and uptake patterns in biological samples, which have been reported in several studies (He et al., 2011; Rodriguez-Gonzalez et al., 2016). Hyphae were also found to have well-developed tube-like structures and cylindrical shapes with smooth surfaces (Figure 12A). SEM images of *A*. *brassicae* showed abundant conidia of long obpyriform in shape (Figures 12A,B). Both the fungicides mancozeb and propiconazole were able to elicit unfavorable alterations in the morphology of *A. brassicae*. Mancozeb-treated fungal wall resulted in collapsed conidia and fewer spores. However, hyphae showed irregular swelling and shrinking, indicating an inflammatory response to fungicides, as shown in Figure 12C. Propiconazole, on the other hand, causes inflated and sunken mycelia with minimum spores observed at a 500 ppm dose (Figure 12D).

After treatment with M-CuO NPs and C-CuO NP, SEM/EDX investigations confirmed the attachment of nanoparticles in the hyphae, which is responsible for the loss of integrity in the hyphal wall causing detrimental shrinkage and affected spore germination (Figures 12E,F). The principal toxicity mechanism is thought to be based on direct physical interactions between nanoparticles and the build-up of reactive oxygen species (ROS). The interaction of both types of CuO NPs provided support for understanding the antifungal mechanism of metal-based nanoparticles (Stabryla et al., 2018). Nonetheless, the antifungal mechanism by which CuO NPs kill fungus is known as the contact killing mechanism to date. All of the behaviors were attributed to the initial direct contact of CuO NPs with fungal spores and hyphae, as observed in compound microscopic and SEM images. Thus, M-CuO NPs have the ability to effectively reduce spore germination, deformed conidia, and hyphae deformity, suggesting that the nanoparticles could be used in the early stages of pathogen infection. Thus, these techniques have convincingly demonstrated a path for the CuO NP antifungal mechanism.

#### 3.10.2 EDX of fungal hyphae

**FIGURE 13:**
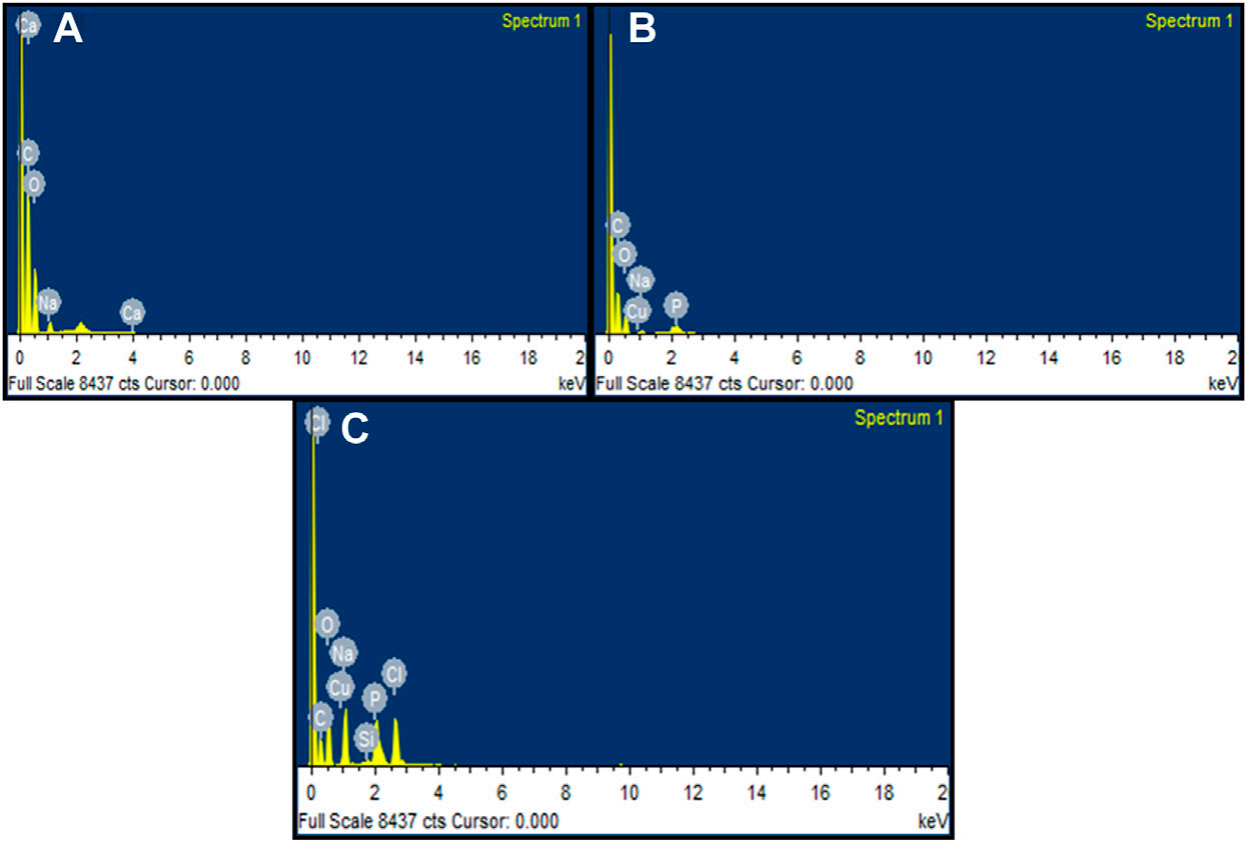
EDX analysis of fungal hyphae: **(A)** control, **(B)** EDX spectra of M**-**CuO NP-treated fungus hyphae, and **(C)** EDX spectra of mycelia treated with C**-**CuO NPs.

EDX spectra identified the presence of elements present in the fungal sample. The presence of peaks such as carbon, oxygen, sodium, and calcium was observed in the control sample containing untreated fungus (Figure 13A). The fungus treated with M-CuO NPs has the presence of elements such as carbon, sodium, phosphorus, oxygen, and copper. Thus, the peak obtained for copper in the fungal sample confirmed the attachment of M-CuO NPs. The presence of 1.36% weight of Cu was recorded in the 100 mg of fungal mycelium treated with M-CuO NPs (Figure 13B). In comparison to the control sample, carbon, sodium, phosphorus, chlorine, silicon, and copper were found in mycelium treated with C-CuO NPs, according to EDX data. Copper was recorded by 0.92% of its weight (Figure 13C).

### 3.11 Transmission electron microscope analysis for the interaction of copper oxide nanoparticles against *A. brassicae*

**FIGURE 14:**
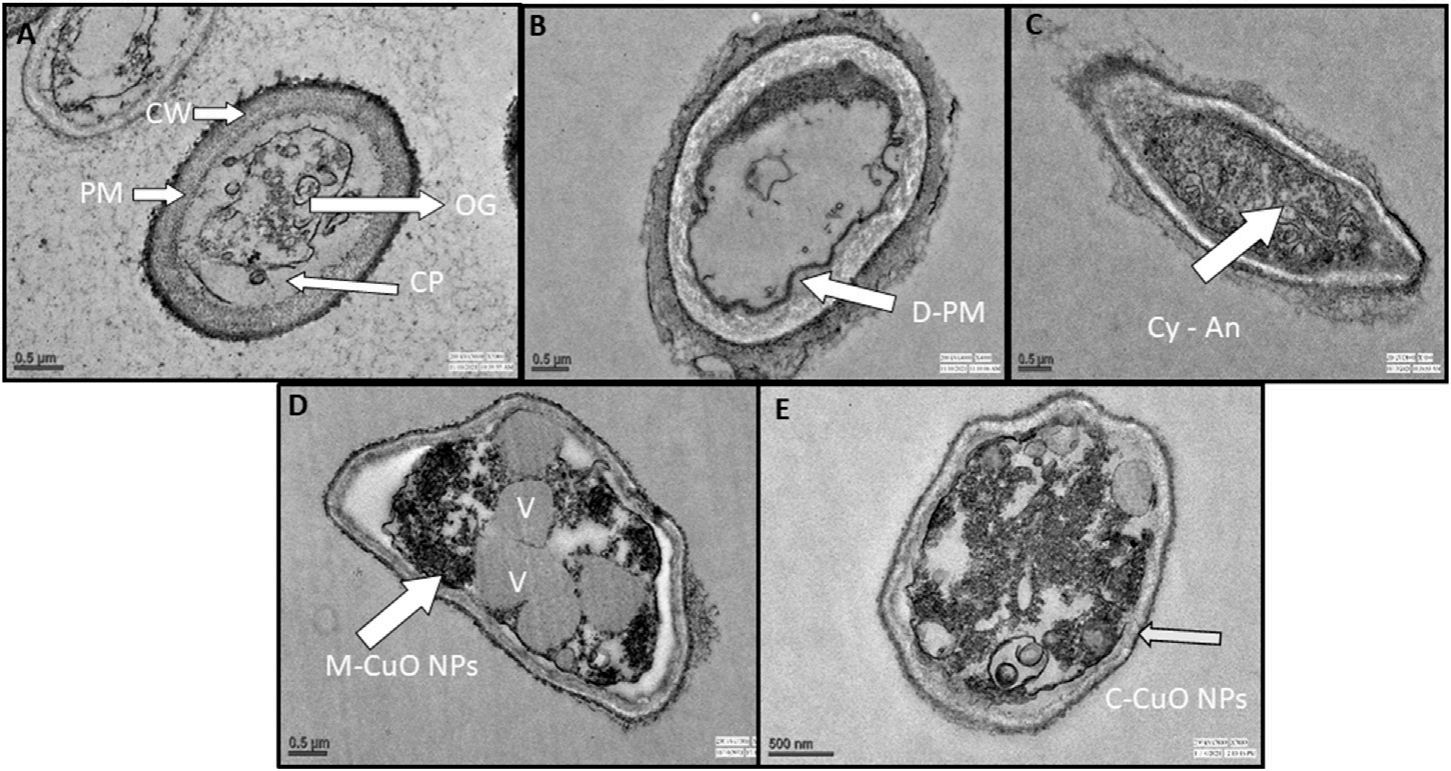
TEM ultrastructure analysis: **(A)** micrographs of *A. brassicae* (control) showing regular cell wall (CW), plasma membrane (PM), cytoplasm (CP), and organelles (OG); **(B)** tested fungi treated with 2000 ppm of mancozeb resulted in delocalization of plasma membranes, cytoplasmic disorder (Cy**-**An); **(C)** propiconazole (0.05%) resulted in the damage of cell wall and hardly recognizable organelles; **(D)** M**-**CuO NPs accumulated inside the cytoplasm, and delocalized cell organelles, leads to large number of vacuoles at 200 ppm in broth assay after 10 days; **(E)** C**-**CuO NPs also entered cytoplasm and creates an imbalance in all organelles, leads to an increased vacuolization.

TEM images of the control sample revealed that fungal hyphae have a normal cell wall, cell membrane, and dense cytoplasm, with clearly visible organelles (Figure 14A). It appears that when the fungicide mancozeb interacts, it permeated within the plasmalemma and caused plasma membrane delocalization. However, substantial effects on the cytoplasm were also observed after treatment with the fungicide mancozeb (Figure 14B). However, the fungicide propiconazole has partially destroyed the cell wall, resulting in an intact cell envelope and hardly recognizable organelles (Figure 14C). In summary, the first stage can be thought to be a local injury to the cell wall, followed by the degradation of the plasma membrane, which involves the inflammatory and sunken response to fungicides. Thus, the mode of action of fungicides is somewhat similar to CuO NPs, as it affects the macromolecules DNA, proteins, and lipids, resulting in cell death.

The interaction of a fungal cell with M-CuO NPs at a 200 ppm dose showed significant effects like high vacuolation, disordered cytoplasm, and hardly recognizable organelles as compared to control. In the cytoplasm, there was a noticeable increase in the aggregation of intracellular vesicles and vacuoles (Figure 14D). The aggregation of CuO NPs in fungal hyphae is thought to cause cytoplasmic dysfunction and other anabolic behaviors. However, after treatment with C-CuO NPs at 200 ppm, significant impacts were detected in fungal cells, including increased vesicle aggregation, disorganized organelles, and accumulation of the nanoparticle in the cytoplasm and nucleus, leading to harmful effects of NPs on hyphae (Figure 14E). In comparison to the effects of fungicides, TEM images of fungal cells treated with CuO NPs showed the irregular and agglomerated CuO NPs distributed in the fungal cell.

### 3.12 Reactive oxygen species-mediated oxidative stress in treated *A. brassicae* with copper oxide nanoparticles

**FIGURE 15:**
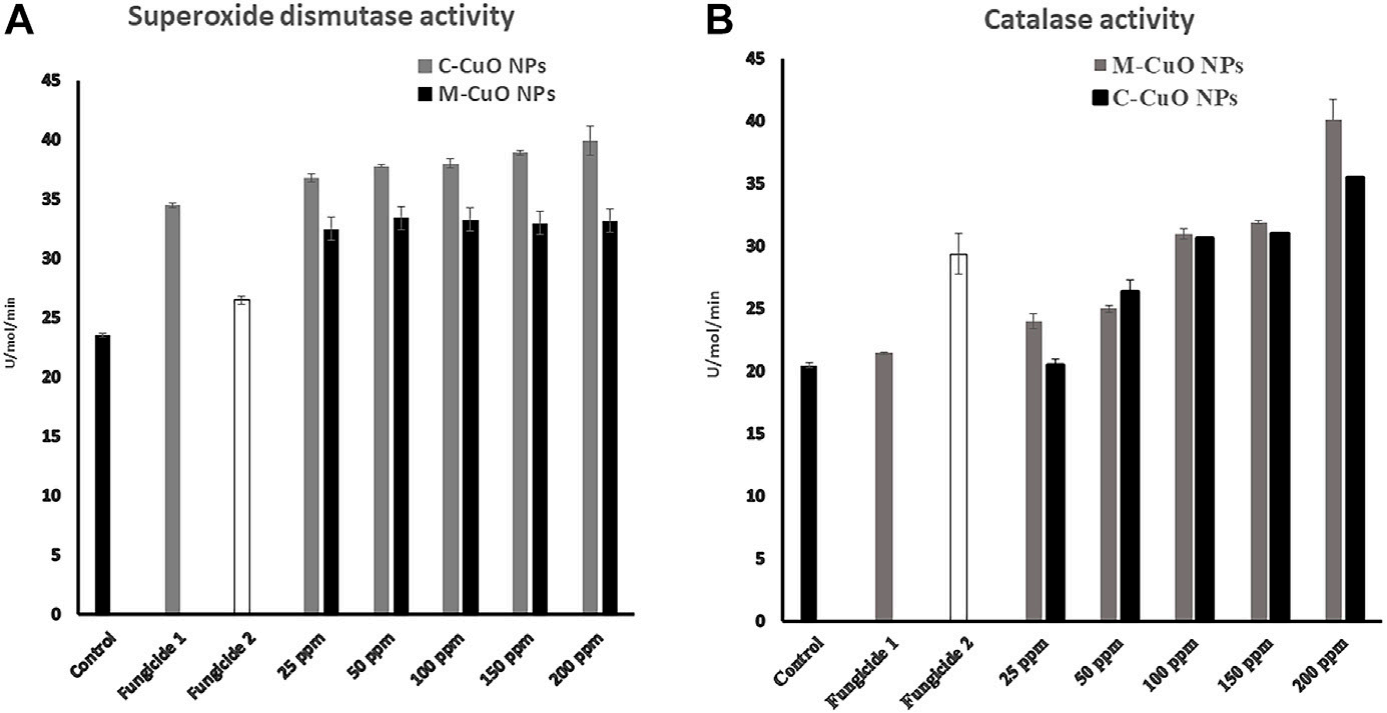
Reactive oxygen species enzyme activation in *A*. *brassicae:* **(A)** superoxide dismutase and **(B)** catalase.

In this study, we measure the SOD by nitroblue tetrazolium (NBT) method that is based on photoreduction (which is a blue-colored formazan) on exposure to light by superoxide radicals. It competes with enzyme SOD for superoxide anions. In the presence of SOD in the reaction mixture, NBT will produce less amount of colored complex than control. Here, we observed that compared to basal ROS levels in control (untreated), 7 days of stressed fungal cells with M-CuO NPs have maximum 39.9 U/mol SOD level, whereas C-CuO NPs, 36.2 U/mol activity of SOD was recorded (Figure 15A). Our results demonstrated that a dose-dependent increase in endogenous ROS levels was significantly elevated by around (58.5%) in the presence of sub-inhibitory concentrations and almost double by MIC (200 ppm). Likewise, there was an increase of around 40.1 U/mol of CAT in M-CuO NP-treated cells, while the increase with C-CuO NPs were evident at 35.5 U/mol (Figure 15B). Thus, one could say that the oxidative stress resulted because of the catalytic action of copper that entered the fungi cells in the form of oxidized atoms delivered by the CuO NP surface. An increased SOD and CAT level indicates the adaptive response of fungi to oxidative stress.

## 4 Discussion

The interdisciplinary field of nanotechnology has been widely used to meet the agricultural demand around the world. Nanoscience leads to the development of low-cost, environmentally friendly nanomaterials, which initiates the disease resistance power of crops exposed to nano-products (Patra and Baek. 2014; Abdelmoteleb et al., 2018). Previous literature studies have reported the various fungal species, *Fusarium*, *Verticillium*, *Alternaria*, *Penicillium,* and *Trichoderma*, employed for nanoparticle synthesis (Hassan et al., 2019; Mohamed et al., 2020; Badawy et al., 2021; Shaheen et al., 2021b). The present study included *T*. *asperellum* for the synthesis of CuO NPs as it is a natural source of secreting the various active molecules, which were antifungal and antimicrobial in nature. The size and morphology of produced nanoparticles were found to be influenced by the reaction mixture temperature, pH, and concentration of salt solution. Moreover, the high temperature adaptation and active metabolites released from the novel microbe *T*. *asperellum* helped in the capping and stabilization of nanoparticles. Cu^2+^ ions were reduced to zero-valent Cu atoms by the presence of enzymes and secondary metabolites (Stracquadanio et al., 2020). These zero-valent Cu atoms initiate the nucleation process by converting the remaining Cu^2+^ to CuO NPs, leading to cluster formation. Moreover, the agglomerated form and smaller sized CuO NPs in the range of (4–12 nm) were found in several studies (Shaalan et al., 2016; Hendi and Rashad, 2018).

In the present study, M-CuO NPs from *T. asperellum* filtrate exhibited a maximum antifungal activity against *A. brassicae*. Remarkably, the maximum inhibition was observed at 200 ppm dose with M-CuO NPs (200 ppm) followed by C-CuO NPs (200 ppm) > mancozeb > propiconazole. The morphological changes observed through compound microscopy at various concentrations suggested that a lower concentration of NPs (150–200 ppm) leads to adverse effects as compared to higher doses of fungicides, which resulted in the deformed hyphal structure and fracture formation. Moreover, irregular swelling and shrinkage, external bulging, and chlamydospore formation were also observed, indicating the leakage of intracellular components. Similar effects have been observed in several fungal species such as *Fusarium solani*, *Neofusicoccum* species, and *Fusarium oxysporum* by the effects of Cu NPs (Pariona et al., 2019). Moreover, in another study by Ouda (2014), CuO NPs have affected the spore germination rate of *A. solani* and *A. alternata*. The antifungal activity of our M-CuO NPs is in agreement with Fatma et al. (2018) who reported the dose-dependent effect of copper complexes on *A. solani* infecting tomato crops under *in vitro* conditions at 500 ppm and proved that Cu NPs were highly effective as compared to Cu (OH)_2_.

SEM and TEM studies further confirmed the deformities observed in the hyphal surface and also showed the attachment of both types of NPs. Our TEM micrographs have proved that both types of CuO NPs were able to enter and found in the agglomerated state; thus, it could be concluded that NPs were absorbed by fungal cells. These results were in agreement with one study, where silver NPs were found to be accumulated on the external cell wall layer and also entered inside the cytoplasm in *A*. *solani* cell (Abdel-Hafez et al., 2016). It could be assumed that CuO NPs produce reactive oxygen species, which changes the normal physiological redox-regulated functions. It has been reported that the damage in cell functions occurred due to reactive oxygen species generated. The protein radicals, DNA-strand breaks, disrupted DNA/RNA, free nucleic acids, and modulation of inflammatory responses through signal transduction have been observed in several leading to cell death and genotoxic effects (Meng et al., 2009; Stankic et al., 2016).

Thus, the interesting wrestle between C-CuO NPs and M-CuO NPs paves the attention of society to synthesize copper oxide nanoparticles that can be applied to manage the phytopathogens. In addition, the metal ions not only interact with cellular proteins and denaturing proteins but also act as essential mineral elements for plant growth. This is because Cu participates in numerous direct and indirect specific physiological and biochemical processes. This leads to metabolic functions of Cu in plant physiology and consequently plays a crucial role in plant defense mechanisms in biotic stress situations. This may contribute to the high biocontrol potential of M-CuO NPs, which can replace standard fungicides. From these perspectives, our study indicates the potential benefits of using M-CuO NPs as a fungicide, which is effective at a lower dose and more stable as compared to chemical fungicides to the fungus proved through *in vitro* plate assay and microscopic studies. Thus, green technology is a promising approach and replacing the traditional fungicides.

## 5 Conclusion

A new initiative for the agricultural applications is the next-generation fungicides and fertilizers; among them, CuO NPs are known to activate the defense system of plants and act as nanofungicides and nanofertilizers. Thus, in the present study, we have synthesized copper oxide nanoparticles from *T. asperellum* culture filtrate and M-CuO NPs were characterized by SEM, TEM, DLS, and XRD. The specific aim of the present study was to check the antifungal activity and mechanism involved for their potential. In the current study, the maximum antifungal activity was at 200 ppm, inhibiting more than 90% growth of tested pathogen *A*. *brassicae.* The chemical fungicides mancozeb and propiconazole inhibited the growth up to 44.2% and 38.7%, respectively.

Light microscopic observations revealed that the conidial length and width were 122.2–136.8 μm and 40–50 μm in the control sample, whereas from M-CuO NPs (50–200 ppm) supplemented media, the conidial length and width were reduced to 122.2–91.0 μm (*p* value < 0.005) and 22.7–16.4 μm (*p* value < 0.005), respectively. However, maximum reduction in spore counts (2 × 10°) was observed with M-CuO NPs at 200 ppm in the short time period of 5 days as compared to the action of fungicides and C-CuO NPs. The results with higher microscopy techniques indicated cytoplasmic dis-functioning, more vacuolization in *A*. *brassicae* cells supplemented with M-CuO NPs. Therefore, higher effects were observed through SEM, TEM, and light microscopy analysis in *A*. *brassicae* cells leading to affect the metabolism and reduced virulence of phytopathogen.

## Data Availability

The datasets presented in this study can be found in online repositories. The names of the repository/repositories and accession number(s) can be found in the article/Supplementary Material.

## Abbreviations

CuO NPs, copper oxide nanoparticles; *A. brassicae*, *Alternaria brassicae*; M-CuO NPs, mycogenic-CuO NPs; C- CuO NPs, chemically synthesized CuO NPs; ppm, parts per million.
