# Supplementary material for: Biocontrol potential of mycogenic copper oxide nanoparticles against Alternaria brassicae
Source: Front Chem. 2022 Aug 30;10:966396. doi: 10.3389/fchem.2022.966396 (PMC9468977; doi:10.3389/fchem.2022.966396)
Supplement: Supplementary file 1 [file DataSheet1.docx]

**Supplementary Table 1: FTIR analysis of Mycogenic CuO NPs and *Trichoderma asperellum* filtrate**

| **FTIR Spectra of M-CuO NPs** | | **FTIR Spectra of Control ( *T. asperellum* )** | | **Reference** |
| --- | --- | --- | --- | --- |
| **Peaks (cm^-1^)** | Types of bond | **Peaks (cm^-1^)** | Types of bond |  |
| **508** | Cu-O | **511** | 0-H, C-Cl, C-Br | (Sankar et al., 2014; Yedurkar et al., 2017; Khatami et al., 2019) |
| **463** | Cu-O | **-** | - | Sulamian et al., 2022 |
| **3396** | N-H | **3348** | N-H | Sulamian et al ., 2022 |
| **2924** | C-H | **2926** | C-H | Ethiraj et al ., 2012 |
| **1600** | C=O | **1541** | C=O,O-H | Suresh et al., 2016 |
| **1467** | N-H | **1649** | C=O | Deepashree et al ., 2013 |
| **1376** | N-H | **1046** | N-H ,O-H | Deepashree et al ., 2013 |

**Supplementary Table 2: Impact of copper oxide nanoparticles on radial growth and percentage inhibition of *Alternaria brassicae***

| **Treatments** | **Radial growth and Percentage inhibition of *A. brassicae*** | | | | | |
| --- | --- | --- | --- | --- | --- | --- |
| **Time of incubation** | **5 DAI** | **Percentage inhibition** | **10 DAI** | **Percentage inhibition** | **15 DAI** | **Percentage inhibition** |
| **Control** | 38.3±0.57^a^ | (0%) | 50.6 ±0.57^a^ | (0%) | 72.3 ±2.5^a^ | (0%) |
| **F1**  **(Mancozeb)** | 29.3±0.57^bc^ | (23.4%) | 35.6 ±0.57^cde^ | (29.6%) | 44.3 ±4.04^b^ | (38.7%) |
| **F2**  **(Propiconazole)** | 28.3 ±0.57 ^bc^ | (26.1%) | 33.3±2.8^def^ | (34.1%) | 40.3±0.5^bc^ | (44.2%) |
| **M-CuO NPs**  **(25ppm)** | 32±4^b^ | (16.4%) | 41±5.2^bc^ | (18.9%) | 43.3±5.5^bc^ | (40.1%) |
| **M-CuO NPs**  **(50ppm)** | 30±4.3^c^ | (21.6%) | 29±9.6^ef^ | (42.6%) | 37±8.1^c^ | (48.8%) |
| **M-CuO NPs**  **(100ppm)** | 28.3±5.8^bc^ | (26.1%) | 27.6 ±4.9^f^ | (45.4%) | 27.6±4.9^d^ | (62.2%) |
| **M-CuO NPs**  **(150ppm)** | 21±5.5^d^ | (45.1%) | 18±6.08^g^ | (64.4%) | 18±6.08^e^ | (75.1%) |
| **M-CuO NPs**  **(200ppm)** | 5.1±0.1^e^ | (86.6%) | 5.1±0.1^h^ | (89.9%) | 5.1±0.1^f^ | (92.9%) |
| **C- CuO NPs**  **(25ppm)** | 37.6±2.08^a^ | (1.82%) | 45.6±1^ab^ | (9.34%) | 45.6±1.1^b^ | (36.9%) |
| **C- CuO NPs**  **(50ppm)** | 37.6±2.08^a^ | (1.82%) | 46±1^ab^ | (9.34%) | 46.1 ± 1 ^b^ | (36.3%) |
| **C- CuO NPs**  **(100ppm)** | 37.3±1.1^a^ | (2.61%) | 39.6±5.5^bcd^ | (21.2%) | 39.6±5.5^bc^ | (45.2%) |
| **C- CuO NPs**  **(150ppm)** | 20.6±0.6^d^ | (46.2%) | 20.6±1.1^g^ | (59%) | 20.6±1.1^e^ | (71.5%) |
| **C- CuO NPs**  **(200ppm)** | 6.03±0.05^e^ | (84.2%) | 8.4±0.3^h^ | (83.3%) | 14.1±0.3^e^ | (80.3%) |
| **CD (p<0.005)**  **(p<0.01)** | 4.562  6.166 |  | 6.991  9.450 |  | 9.224  6.8369 |  |
| **CV** | 10.139 |  | 13.506 |  | 11.661 |  |

***Data representing the radial growth of *Alternaria* *brassicae* in mm as average growth of three replicates ± Standard deviation of mean**

**CD= Critical difference**

**CV =Coefficient of variance**

**DAI= Days after incubation**

**Supplementary Table 3: Antifungal activity of CuO NPs**

| **CuO NPs** | **Source of production** | **Target organisms** | **Effective Concentration** |
| --- | --- | --- | --- |
| B-CuO NPs | *Malus domestica.* | *Lacio diplodia* and *Aspergillus niger* | 25 mg/L |
| B-CuO NPs | Papaya leaf extract | *Ralstonia solanacearum* | 250 mg/L |
| C-CuO NPs | Chemically Synthesized | *Alternaria solani, Fusarium oxysporum, Clavibacter michiganensis, Pseudomonas syringae* | 1000 mg/L |
| B-CuO NPs | *Eicchornia* | *Fusarium culmorum* and *Aspergillus niger* | 100 mg/L |
| B-CuO NPs | *Azadirachta indica* | *Alternaria mali,* *Diplodia seriata* and *Botryosphaeria dothidea* | 1000 mg/ml |
| B-CuO NPs | *Trichoderma harzianum* | *Alternaria alternata, Pyricularia oryzae* | 20 ppm |
| B-CuO NPs | *Penicillium chrysogenum* filtrate | *Fusarium oxysporum*, *Aspergillus nige*r, *Penicillium citrinum*, *Erysiphe cichoracearum* and *Alternaria solani* | 250 mg/L |
| C-CuO NPs | Cas. No. 544868 | *Colletotrichum gloeosporioides* | 500 mg/ml |
| C-CuO NPs | Chemically purchased | *Botrytis cinerea*, *Alternaria alternata* and *Monilinia fructicola* | 310 mg/ml) |
| C-CuO NPs | Hydrothermally  (Tween 20) | *Phytopthora infestans* | 1000mg/L |

**Supplementary Table 4: - Average dimensions and septation number of conidia after treatment with M-CuO NPs and C-CuO NPs**

| **Treatments** | **Spore counts**  **(Spores /1 ml of suspension)** | **Conidial Length**  **(** µm) | **Conidial**  **Width**  **(** µm) | **Horizontal**  **Septa** | **Vertical**  **Septa** |
| --- | --- | --- | --- | --- | --- |
| **Control** | 4x 10 ^2^ | 122.2-136.8 | 40.0-50 | 8-9 | 2-3 |
| **F- 1 (Mancozeb)** | 4x10^0^ | 96.3- 95.1 | 26.0±0.24 | 1-3 | 1-2 |
| **F- 2 (Propiconazole)** | 3x10^1^ | 96.24-94.3 | 23.8±0.47 | 1-3 | 1-2 |
| **M- CuO NPs (25 -200 ppm)** | 3x10^2^_,_ 2x10^1^_,_ 1x10^1^_,_ 4x10^0^  2x10^0^ | 122.3-91.0 | 22.7-16.7 | 4-5 | 1-2 |
| **C- CuO NPs (25 -200 ppm)** | 4x10^2^_,_4x10^2^_,_4x10^1^ _,_5x10^0^_,_ 5x10^0^ | 105.9-99.3 | 36.0- 35.2 | 4-5 | 2-3 |
